# Immunogenicity and Safety of the New Inactivated Quadrivalent Influenza Vaccine Vaxigrip Tetra: Preliminary Results in Children ≥6 Months and Older Adults

**DOI:** 10.3390/vaccines6010014

**Published:** 2018-03-08

**Authors:** Emanuele Montomoli, Alessandro Torelli, Ilaria Manini, Elena Gianchecchi

**Affiliations:** 1 VisMederi srl, Strada del Petriccio e Belriguardo, 35, 53100 Siena, Italy; montomoli@vismederi.com (E.M.); torelli@vismederi.com (A.T.); 2 Department of Molecular and Developmental Medicine, University of Siena, 53100 Siena, Italy; ilaria.manini@unisi.it; 3 Department of Life Sciences, University of Siena, 53100 Siena, Italy

**Keywords:** Vaxigrip Tetra, Influenza, Influenza vaccines

## Abstract

Since the mid-1980s, two lineages of influenza B viruses have been distinguished. These can co-circulate, limiting the protection provided by inactivated trivalent influenza vaccines (TIVs). This has prompted efforts to formulate quadrivalent influenza vaccines (QIVs), to enhance protection against circulating influenza B viruses. This review describes the results obtained from seven phase III clinical trials evaluating the immunogenicity, safety, and lot-to-lot consistency of a new quadrivalent split-virion influenza vaccine (Vaxigrip Tetra^®^) formulated by adding a second B strain to the already licensed TIV. Since Vaxigrip Tetra was developed by means of a manufacturing process strictly related to that used for TIV, the data on the safety profile of TIV are considered supportive of that of Vaxigrip Tetra. The safety and immunogenicity of Vaxigrip Tetra were similar to those of the corresponding licensed TIV. Moreover, the new vaccine elicits a superior immune response towards the additional strain, without affecting immunogenicity towards the other three strains. Vaxigrip Tetra is well tolerated, has aroused no safety concerns, and is recommended for the active immunization of individuals aged ≥6 months. In addition, preliminary data confirm its immunogenicity and safety even in children aged 6–35 months and its immunogenicity in older subjects (aged 66–80 years).

## 1. Introduction

**Figure 1:**
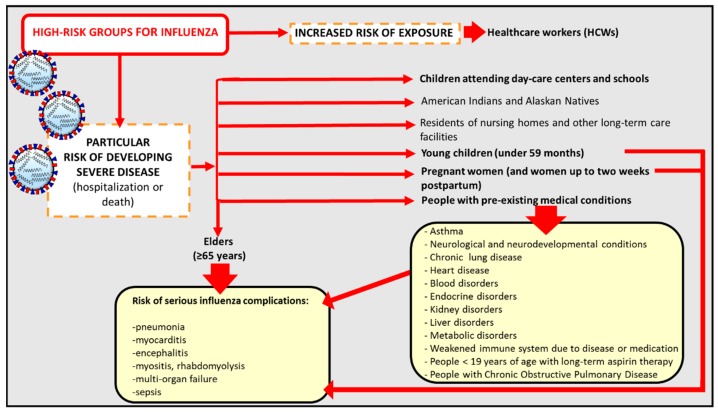
High-risk groups for influenza and influenza-related complications.

Influenza is an acute viral respiratory disease caused by RNA-enveloped viruses which belong to the family of *Orthomyxoviridae* and are transmitted principally through aerosols and droplets generated by coughing and sneezing by infected people. Influenza viruses are responsible both for seasonal influenza epidemics, especially in temperate climates, with a peak during the winter, and sporadic out-of-season cases and outbreaks. In tropical regions, influenza outbreaks are more irregular and influenza cases occur throughout the year [1]. Influenza involves 3–5 million people every year worldwide [2] especially during the winter season [3]. It usually causes fever and may be associated with headache, fatigue, muscle and joint pain, coughing, chills, and runny nose; however, it can also lead to serious illness, such as myositis, pneumonia, encephalitis, multi-organ failure, and fulminant disease in children [4]. Influenza is responsible for both excess hospitalization and mortality; with between 250,000 and 500,000 influenza-related deaths every year, it places a significant economic burden on healthcare services [5,6]. These data emphasize the importance of vaccine prophylaxis. As life expectancy increases, and hence the proportion of elderly people, the number of people suffering from chronic illnesses is growing. Although influenza causes morbidity across the age-spectrum, it is the elderly who are most vulnerable to its serious complications, such as pneumonia [7,8]. In addition to the elderly, other groups with a higher susceptibility to developing severe influenza and its complications (indicated as hospitalization or death) are young children, people with pre-existing comorbidities (such as asthma, chronic lung disease, heart disease, blood and endocrine disorders (diabetes mellitus)), and pregnant women [9]. This last group is deemed by the World Health Organization (WHO) to have the highest priority for influenza vaccination, since contracting influenza during pregnancy is hazardous. Indeed, pregnant women who develop influenza illness are at risk of adverse pregnancy outcomes. Moreover, infants up to the age of 6 months cannot be vaccinated on account of their age. Other subjects at risk of contracting influenza are those who are frequently exposed to the virus, such as healthcare workers (HCWs) [10]. Notably, a group that plays a significant role in the transmission of the virus within the community is that of children attending day-care centers and schools (Figure 1) [11].

Two influenza virus subtypes can cause infection in humans: type A and type B. The former causes the highest number of seasonal influenza infections; in addition to humans, also domestic animals, such as pigs, birds, and horses, can constitute the reservoir for the virus, wherein re-assortment events can occur. More specifically, re-assortment among different influenza A viruses can occur in the case of co-infection. In this case, a virus endowed with novel antigenic properties could cause pandemics if it is able to replicate in humans. Pandemics have been described since the middle of the 18th century, and occur at intervals of 10–40 years [12]. Influenza B viruses is common in children, young adults, and the elderly, and causes seasonal epidemics every 2–4 years. However, limited data regarding its impact in adults and its clinical burden are currently available. It is characterized by a slow mutation rate. While it can occasionally infect seals, it does not have a domestic animal reservoir [13]. Influenza A and B are not clinically distinguishable [14]; however there is a general conviction that the former is responsible for more serious influenza cases than the latter. This is probably why researchers have seldom focused on influenza B, as is demonstrated by the scarcity of data on its epidemiology and burden [15].

Influenza is a vaccine-preventable disease, and vaccines constitute the most effective strategy for the prevention and control of seasonal influenza virus infections and for reducing influenza-related morbidity and mortality [16]. The elevated rate of mutation that characterizes influenza viruses could allow the emergence of new antigenic variants with the ability to evade the immunity induced by previous vaccinations or infection processes, thereby facilitating transmission among humans [17]. For this reason, in order to ensure matching between the viruses most likely to circulate in the upcoming season and the strain present in the vaccine, the process of selecting the viral strain to be included in the seasonal vaccine is performed by the WHO on the basis of information on human influenza virus epidemiology provided by the Global Influenza Surveillance Network (GISN). This process has been carried out since 1971, and in 1998 its frequency was increased from once to twice per year, in order to provide separate recommendations for each hemisphere (in February in the northern hemisphere [18] and in September in the southern hemisphere [19]). These recommendations are issued about 9 months before the vaccine is administered to the population, in order to allow enough time for the manufacture and distribution of the vaccine. Significant efforts have been made by the WHO Global Influenza Surveillance and Response System (GISRS) and its partners to improve this process of assessment [20]. Until 1978, seasonal influenza vaccines included only two strains (one of type A influenza and one of type B); since then, vaccine complexity has increased. Two influenza type A subtypes (A/H1N1 and A/H3N2 strains) and one B strain (belonging to B/Yamagata or B/Victoria lineages) constitute the traditional seasonal influenza vaccines, defined as Trivalent influenza vaccines (TIVs) [21]. Currently, TIVs account for the majority of seasonal influenza vaccines.

Since 1985, two antigenically distinct phylogenetic lineages (the Victoria and Yamagata lineages) [22] have diverged from influenza B viruses. Until that year, a single influenza B lineage was present globally, which was the precursor of the Yamagata lineage.

By 1975, the Victoria lineage had developed in China from a minor lineage of B viruses, although it was not isolated outside the country until 1985 [23]. From 1987 to 1989, it was the prevalent lineage worldwide, and re-emerged in 2001–2002. From then on, it has co-circulated globally with the Yamagata lineage, as in the US since 2001 [24], in variable and unpredictable proportions among regions and seasons [25]. During the 2007–2008 influenza season, when the B viruses co-circulated worldwide, higher mortality rates were reported in the US, as were excess hospitalization rates in children between 0 and 4 years of age [26]. Recently, influenza B viruses have constituted a significant cause of flu, accounting for about 23% of the circulating strains around the world and even reaching 90% during some seasons, thus contributing significantly to influenza morbidity [27]. In each influenza season, one of the two B lineages may be prevalent over the other, making it very difficult to predict the predominant B lineage to be included in the next seasonal vaccine [26,28]. The terms match/vaccine match and mismatch/vaccine mismatch are related to the level of similarity or difference among the predominant circulating lineage and the strains included in the vaccines. In the case of vaccine mismatch, this evolution of influenza viruses has reduced vaccine protection, owing to the limited or absent cross-lineage protection elicited by TIVs [29].

It has been estimated that, between 2000 and 2013, discrepancies between the circulating B lineage and that present in the vaccine formulation occurred in about one-quarter of seasons in which influenza B caused more than 10% of influenza cases [27,30,31,32]. Moreover, eventual cross-protection is estimated to be low in the event of mismatch, owing to the great antigenic difference between the two influenza B lineages [33]. For instance, the efficacy of the B lineage vaccine, which is predicted to be 71–77% when circulating influenza B and B vaccine strains match, drops dramatically (46–49%) in the case of mismatch, significantly limiting the protective effects of TIVs [34], mainly in high-risk groups, such as the elderly, as reported by the group of De Jong [35] during the major epidemic A(H3N2) virus strain.

These data underscore the need to broaden protection by using quadrivalent influenza vaccines (QIVs) [25], as was proposed by the US FDA in 2007 [36]. Since 2012, QIVs have been commercially available in various formulations (such as split, subunit, and live attenuated influenza (LAIV) formulations) and are substituting the corresponding TIV composition throughout the world. QIV overcomes the risk of low antigenic matching between the vaccine strains and circulating viruses, since it contains two influenza B strains and may confer greater protection than the traditional TIV. This could allow both hospitalization and mortality to be reduced [33] and, even if the cost of QIV is higher than that of TIV, replacement of the latter would be significantly cost-effective both for society and for third parties [37]. This result has been confirmed by a study by the group of de Boer, which estimated the substantial cost-effectiveness of replacing TIV with QIV, with a net societal budget impact of $5.8 billion in the US and a 27.2% reduction in influenza cases caused by B lineages in the next two decades [38]. QIVs were first introduced in the US [39], and subsequently became part of the immunization strategies of several countries. Concerning the US, during the current influenza season (2017–2018) both TIV and QIV are available, and the Advisory Committee on Immunization Practices (ACIP) recommends annual vaccination for all subjects ≥6 months of age who do not have contraindications. By contrast, it does not recommend the use of LAIV (administered as a nasal spray flu vaccine), owing to concerns regarding its effectiveness against (H1N1)pdm09 viruses during the 2013–14 and 2015–16 seasons [40].

## 2. Evaluation of Influenza Vaccine Efficacy: From CHMP Criteria to Post-CHMP Criteria

The efficacy of influenza vaccines is traditionally evaluated by means of serological assays that identify influenza-specific serum antibodies induced by the vaccine itself as correlates of protection [41]. These consist of the single radial hemolysis assay (SRH) [42] and hemagglutination inhibition assay (HI) [43].

The SRH assay enables antibodies to be quantified by measuring hemolysis areas [44,45], which are proportional to the antibody concentration, since they result from the binding between the antigen and the antibody. The assay is rapid, easily reproducible, and reliable, and also allows a high number of samples to be analyzed; for this reason, it is notably appropriate for large-scale studies, particularly epidemiological investigations [46,47].

The HI assay is also a relatively simple test to automate. It is based on erythrocyte agglutination due to the binding of the viral surface protein hemagglutinin (HA) to sialic acid sites on the surface of red blood cells, thereby ensuring the internalization of the virus. The HA content is correlated with the number of agglutinated erythrocytes [44]. The erythrocytes commonly used are of avian origin (chicken or turkey) or mammalian origin (horse or guinea pig). HI titers are quantified as the reciprocal of the highest serum dilution (titer) (1/dilution factor) that inhibits hemagglutination by binding with the virus. Hemagglutination is inhibited or blocked when the antibodies recognizing HA are present in the serum in a sufficient number [48]. One drawback to both SRH and HI is that they are not standardized tests, and display significant inter-laboratory variability; for this reason, in clinical development programs, both tests should be performed in designated and properly qualified laboratories [49].

The microneutralization (MN) assay, also known as the Virus Neutralization (VN) assay, is not compulsory for influenza vaccine licensure. However, it is strongly recommended that it should be performed at least in a representative subpopulation of clinical trial participants, if not in all. MN detects the antibodies capable of neutralizing the ability of the virus to enter or replicate in mammalian cells. The MN assay also identifies those antibodies that recognize neuraminidase (NA). For this test, the antibody titer is expressed as the reciprocal of the highest serum dilution that causes at least 50% of cytopathic effect in mammalian cell cultures. Madin–Darby canine kidney (MDCK) cells are usually used [50]. Although MN is an adequate substitute for HI, the best protocol for performing the MN test has not yet been established. Whereas no correlates of protection have yet been established for MN, an SRH titer of ≥25 mm^2^ and an HI titer of ≥1:40 are considered to be associated with 50% or higher protection against influenza [46] and represent the thresholds that have to be met for licensing of the vaccine in Europe [51].

Three criteria have been identified by the Committee for Medicinal Products for Human Use (CHMP). The percentage of vaccines presenting an HI titer ≥ 40 or SRH > 25 mm^2^ should be >70% in subjects aged 18–60 years and >60% in the over-60s. The seroconversion rate (SCR) (at least a 4-fold increase in titer) should be >40% in 18–60-year-olds and >30% in the over 60s. A mean geometric increase (ratio of pre- to post-vaccination) of >2.5 is required in 18–60-year-olds and >2 in over-60s. Regarding the US, the FDA applies the same criteria, but the lower bound of the 95% confidence interval (CI) must be higher than or equal to that of the SCR and geometric mean titer (GMT) criteria (Table 1) [52].

At least one of the criteria mentioned above has to be met for the annual licensing of seasonal vaccines and all three criteria for pandemic vaccines. However, these criteria present limitations. They were established decades ago on the basis of a limited number of investigations performed in healthy adults, whereas high-risk groups, such as children, were not included in these evaluations.

The neonatal immunological response to vaccines is weak; in more detail, the neonatal immune system is biased against Th1 responses, supposedly to prevent a pro-inflammatory immune response towards the maternal tissues that could cause preterm birth or spontaneous abortion [53]. Furthermore, newborns show an impaired humoral response responsible for a qualitatively and quantitatively lower antibody production respect to older infants and CD4 lymphocyte responses are often weaker and less sustained than those of adults [54]. Concerning the immune response upon influenza vaccination in children, a correlation between an increased antibody production and age has been reported [55]. Furthermore, conversely from vaccinated adults, children are characterized by poor cross-reactivity in case of influenza B virus vaccine-mismatch [56].

Regarding the elderly, a progressive decline in the humoral and cellular immune responses (a phenomenon also referred to as “immunosenescence”) towards infections and vaccinations is observed [57]. Several extrinsic (such as co-morbidities and stress) and intrinsic (genetic background) factors can exert an effect on it [58]. In the elderly, the vaccine immune response is diminished respect younger adults [59] with an effectiveness reduced of 50% also in presence of elevated influenza vaccination rate and well-matched vaccine [60,61]. Subjects with a reduced health status present a defective number and function of Natural Killer (NK) cells that are positively associated with low HI titers in vaccinated older subjects [62,63]. In addition, older individuals show a defective differentiation and function of B and T lymphocytes, reduced antibody production as well as costimulatory molecule CD28 expression on T lymphocytes [64,65,66,67,68].

The evaluation of the efficacy of LAIV cannot be properly performed through serological assays. This has led to changes in the assessment of influenza vaccines in Europe since 2014 [69]; these have promoted a more global view of vaccine immunogenicity, which includes not only the evaluation of HI titers, but also the evaluation of MN titers and techniques used for the quantification of cell-mediated immunity (CMI), through flow cytometry. CMI is performed in a randomly selected subset of the subjects included in the clinical trial, and is encouraged especially in the elderly (≥75 years old) since it is associated with the establishment of protection related to humoral immunity. Indeed, although the elderly may present higher HI and MN antibody titers than young adults, these titers might not be correlated with protection [70].

The advantage of flow cytometry is that it can evaluate vaccine efficacy by simultaneously characterizing lymphocyte phenotype and responses upon vaccination in a heterogeneous cell population. This is achieved through the use of fluorescent-labeled antibodies to detect specific proteins that can be expressed on the surface of the cells or intracellularly. It is recommended that T-cell immune responses be investigated as well as the activation of CD4, CD8, and memory B lymphocytes, since they can provide more information regarding both immune responses and protection elicited by influenza vaccination. However, the choice of which populations to analyze is not simple. Moreover, no correlates of protection concerning cell populations following vaccination are yet available and results can be difficult to interpret [71].

Other widely used methods of evaluating lymphocyte functions are ELISA [72] and ELISPOT [73] for the detection of cytokine responses, ^3^H-thymidine incorporation or carboxyfluorescein succinimidyl ester CFSE for the assessment of T-cell proliferation upon encountering an antigen, chromium release assay for the determination of antigen-specific cytotoxicity, and the investigation of differential gene or microRNA expression. In recent years, several new T-lymphocyte methods, including the improved FLUOROSPOT ELISPOT assay, and novel techniques such as cytometry by time-of-flight mass spectrophotometry (CyTOF) have been developed. Whereas the former is able to detect multiple cytokines in the same well, the latter assesses more than 50 parameters concurrently. Currently, despite the availability of several assays for the assessment of cellular immunity, no correlates of protection have so far been established for these techniques, which, in addition, display marked inter-laboratory variability, not least because of the absence of standardized protocols [3].

## 3. General Information on QIV

In recent years, the efficacy of several QIVs has been investigated. The group of Domachowske [74] reported that QIV was not less immunogenic than TIVs in children aged from 3 to 17 years. TIVs have proved able to induce some degree of cross-reactivity against B strains, though less antibody production has been observed for the unmatched strains than for the matched strains. Furthermore, for the unmatched strains, all EMA criteria have not been met by TIVs [33,75,76,77].

## 4. A Novel Quadrivalent Inactivated Influenza Vaccine: Vaxigrip Tetra

Vaxigrip Tetra^®^ (Sanofi Pasteur) is a novel four-strain influenza vaccine belonging to the Vaxigrip^®^ family of influenza vaccines [78]. It is a quadrivalent split-virion inactivated influenza vaccine containing the purified HA and NA antigens from the four influenza virus strains recommended by the WHO [79]. The total amount of HA glycoprotein contained in Vaxigrip Tetra is 60 µg. The viral growth of the strains constituting Vaxigrip Tetra, as well as the corresponding TIV (Vaxigrip^®^, Sanofi Pasteur, Lyon, France) formulated with two A strains (A/H1N1 and A/H3N2) and a single B strain and containing a total amount of 45 µg HA glycoprotein, is conducted in embryonated chicken eggs. Following the collection, clarification, and concentration of the allantoic fluid, the viral particles are purified and split by means of the detergent octoxynol-9. Finally, inactivation by means of formaldehyde is performed in order to obtain 4 monovalent bulks for each strain, which are formulated into a sterile suspension of phosphate buffered saline without preservatives [80].

### 4.1. Clinical Trials Performed on Vaxigrip Tetra

The immunogenicity and safety of Vaxigrip Tetra have been investigated in six phase III clinical trials in subjects aged ≥3 years and in one recent phase III clinical study conducted on children aged 6–35 months (Table 2). Three of these studies were considered pivotal investigations, since their aim was to characterize Vaxigrip Tetra immunogenicity for approval in the EU [81,82,83]. The licensed Vaxigrip Tetra formulated through a revised process was evaluated by these three main trials [81,82,83] and by another involving adults aged 18–60 years [84]. Two phase III clinical trial were considered supportive; these were conducted on adults, the elderly and children from 9 years of age, and investigated Vaxigrip Tetra batches produced with the same method as Vaxigrip [85,86]. A further randomized double-blind placebo-controlled trial involving children aged from 6 to 35 months was concluded recently, and preliminary data concerning this group are available [87]. The safety profile was investigated collecting unsolicited adverse events (AEs) and serious adverse events (SAEs) in accordance with International Conference on Harmonization E2A Guideline for Clinical Safety Data Management: Definitions and Standards for Expedited Reporting [88]. The term AE indicates “*any untoward medical occurrence which occurs during administration of a vaccine or follows immunization and which does not necessarily have a causal relationship with the use of the vaccine*”. The AE could be any unfavorable or unintended sign, an abnormal laboratory finding, a symptom or a disease”. The term SAE indicates “*A serious adverse event (experience) or reaction is any untoward medical occurrence that at any dose results in death or is life-threatening”* [88]. SAEs have to be compulsorily notified [89].

#### 4.1.1. GQM01 Clinical Trial in Adults

Pépin and coll. [85] conducted a phase III, randomized, active-controlled, multicenter trial in Germany and France (GQM01; EudraCT No. 2011-001976-21) in two subgroups of healthy adults (18–60 and >60 years) during the 2011/2012 influenza season. The aim of the study was to demonstrate the non-inferiority of QIV in inducing antibody production in comparison with the 2011–2012 licensed TIV (containing the B/Brisbane/60/2008 strain, belonging to the Victoria lineage) and an investigational TIV containing the alternative B strain lineage (B/Florida/04/2006, Yamagata lineage). The secondary objective was to evaluate the safety profile of the vaccines, including potentially SAEs. AE of special interest (AESI) were represented by anaphylaxis, Guillain–Barré syndrome, encephalitis, myelitis, neuritis, convulsions, and vasculitis and, as well as AEs, were classified by the investigator as related or unrelated to vaccination. Secondly, the putative superiority of B-strain immune responses induced by QIV in comparison with TIV not containing the matched B strain was also investigated. To this end, 1116 subjects were vaccinated with QIV, 226 with the licensed TIV, and 223 with the investigational TIV. HI titers were evaluated pre-vaccination and 21 days post-vaccination; in all groups, comparable pre-vaccination HI antibody titers were observed which increased upon vaccination. In comparison with TIV, QIV was able not only to induce non-inferior antibody responses against all four matched strains, but also to promote greater antibody production than the TIVs lacking the corresponding B strain for both B strains in both age-groups. For all four vaccine strains, QIV met EMA criteria in both age-groups. Furthermore, for the three vaccines, the younger age-group generally showed higher post-vaccination GMT ratios and response rates than elderly adults [85]. This finding is in agreement with other studies, and is ascribed to the waning responsiveness of the elderly immune system and to other age-related factors [90,91]. Concerning TIVs, EMA criteria were met for the A strains and the matched B strains, but not for the unmatched B strains.

Regarding safety, similar solicited reactions (usually injection-site pain, headache and myalgia resolved within 3 days of vaccination), unsolicited AEs, and SAEs were reported in the QIV and pooled TIV groups. Nasopharyngitis (1.1% vs. 0.9% for subjects immunized with QIV and TIV, respectively) and injection-site pruritus (1.3% vs. 1.1% for individuals receiving QIV and TIV, respectively) constituted the most common vaccine-related AEs. Neuritis was the single AESI reported in a TIV vaccinated elderly subject, but it was classified as not treatment-related. No vaccine-related SAEs or deaths were observed [85].

#### 4.1.2. GQM04 Clinical Trial in Children, Adolescents, and Adults

A phase III randomized, controlled, multicenter trial conducted in Australia and in the Philippines in 2012 (GQM04; NCT01481454) investigated the safety, immunogenicity and lot consistency of Vaxigrip Tetra in children/adolescents (9–17 years, *n* = 329) and adults (18–60 years, *n* = 1648) in comparison with licensed TIV (children/adolescents *n* = 55; adults *n* = 56) [86]. The study recruited children, adolescents, and adults who had not been immunized with influenza vaccine containing the 2012 Southern Hemisphere formulation or a 2011–2012 Northern Hemisphere formulation within 6 months before enrollment or any other vaccine during the previous 4 weeks. Investigation of immunogenicity (evaluated by means of HI and MN) and safety profiles confirmed the comparability of QIV and TIV, in agreement with previous results [85]. EMA criteria were met in adult groups for all four strains contained in QIV; in addition, the antibody response seen in the group of children/adolescents was superior to that recorded in adults, though EMA criteria have not yet been established for the former.

In addition, QIV lot-to-lot equivalence among the three lots examined for all four strains and in both age-groups was demonstrated, supporting the conviction that Vaxigrip Tetra can be produced consistently across seasons and producers [86]. Equivalent solicited reactions, unsolicited AEs, and SAEs were observed between TIV and QIV recipients in both age groups. Vaccine-related SAEs or deaths were not reported [86].

#### 4.1.3. GQM02 Clinical Trial in Children (3–8 Years)

A subsequent phase III randomized, double-blind, active-controlled, multi-center study conducted in Finland, Poland, Mexico, and Taiwan on 1242 children aged 3 to 8 years during the 2013/2014 Northern Hemisphere influenza season (GQM02; EudraCT No. 2011-005374-33) investigated the immunogenicity and the safety profile of Vaxigrip Tetra (which contained the 4 Northern Hemisphere 2013/2014 influenza strains recommended by the WHO and the EU) in comparison with its trivalent counterpart 2013–2014 formulation (TIV-1, Vaxigrip^®^), which contained both A strains and the B Victoria lineage strain, and an investigational TIV (TIV-2) containing both A strains and the B Yamagata lineage strain [82]. HI titers at the baseline (indicated as day 0) and 28 days after the last vaccination were measured in order to evaluate antibody responses induced by QIV and by TIVs. The acceptability of the QIV safety profile and the non-inferiority of this vaccine to TIV for the shared strains were confirmed. In addition, it was observed that QIV displayed higher immunogenicity towards influenza B strains than a TIV formulated with the alternative B strain lineage. In detail, although the HI antibody titers were relatively high at the baseline, probably as a consequence of natural exposure to the same strains or to one similar to those contained in the QIV formulation, GMTs were enhanced at least 6-fold for all vaccine strains upon QIV immunization [82]. Seroprotection against all the strains was achieved in almost all vaccines [82], whereas seroconversion rates were similar to those found by Cadorna [86] in children and adolescents aged from 9 to 17 years, ranging from 64.8% to 88.5% for all four strains [82].

Regarding the safety profile, 62.4% of QIV and TIV recipients notified solicited injection-site reactions; pain was the most frequently reported solicited reaction in both groups. Regarding solicited systemic reactions, they were reported by 48.9% and 45.5% of subjects immunized with QIV and TIV, respectively, with malaise representing the most common solicited systemic reaction, followed by myalgia and headache. No significant difference was observed in the rates, types, or severity of solicited reactions between QIV and TIV groups, and they were classified as grade 1 (mild) and resolved within 3 days or less by their onset. An unique AESI of severe thrombocytopenia was observed 9 days after a first vaccination in one 3-year-old subject receiving QIV and it resolved after 38 days; it was considered vaccine-related due to the temporal relationship with the immunization [82].

#### 4.1.4. GQM09 Clinical Trial in Children and Adolescents

The aim of the open-label phase III study (GQM09; U1111-1127-7693) was the assessment of the immunogenicity (evaluated through HI) and safety of the 2013–2014 Northern Hemisphere QIV formulation in 100 children and adolescents 9–17 years of age enrolled in Taiwan [81]. Although pre-vaccination titers were relatively high, post-vaccination HI titers enhanced for all four strains. Following vaccination, seroprotection rates reached 99% for A/Texas/50/2012 (H3N2) and 100% for A/California/7/2009 (H1N1) pdm09, B/Massachusetts/2/2012, and B/Brisbane/60/2008. Rates of seroconversion/significant increase of HI titer were relatively low due to the elevated pre-vaccination titers. The most common solicited reactions were injection-site pain (56%), myalgia (45%), and malaise (15%) which in general were mild or moderate. No treatment-related AEs, immediate unsolicited AEs, unsolicited non-serious injection-site AEs, grade 3 unsolicited AEs, or SAEs were notified. The present clinical trial demonstrated that QIV was immunogenic and well tolerated [81].

#### 4.1.5. GQM11 Clinical Trial in Younger and Older Adults

The aim of a pivotal multicenter phase III study (GQM11; EudraCT No. 2014-000785-21) [83] was to evaluate the safety, immunogenicity (through HI and serum neutralization assays on D0 and D21) and lot-to-lot consistency of the 2014–2015 Northern Hemisphere formulation of Vaxigrip Tetra in comparison with a licensed TIV containing B Yamagata and an investigational TIV containing B Victoria. The study involved a total of 2225 subjects (1114 adults (18–60 years) and 1111 subjects (>60 years)) in three centers in Belgium, three in France, four in Germany, and five in Poland from September 2014 to October 2015 [83]. More in detail, 1024 subjects (18–64 years), 629 (65–84 years), and 13 (over 85 years) were immunized with QIV; whereas 348 (18–64), 201 (65–84), and 4 (over 85) received TIV [92].

**Figure 2:**
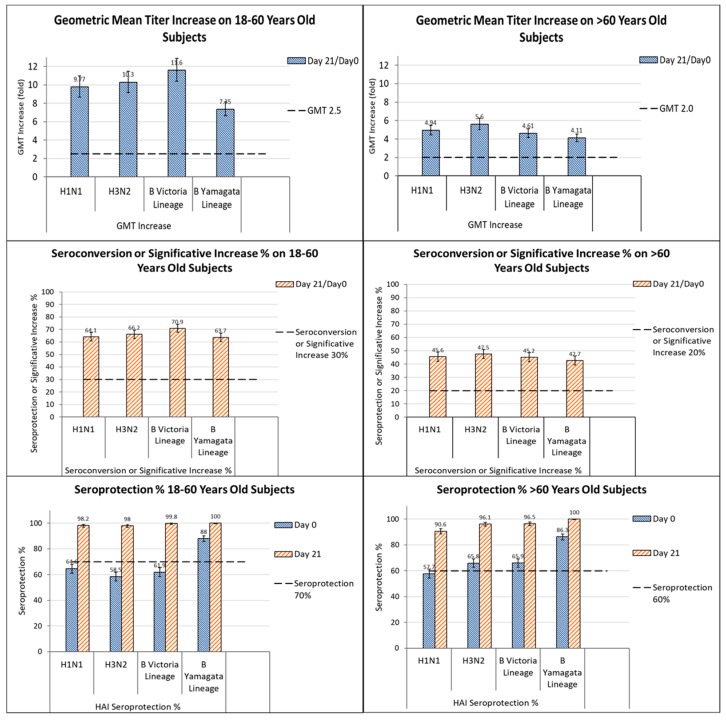
GMT increase, seroconversion or significant increase % and seroprotection % induced by Vaxigrip Tetra in subjects aged 18–60 years and >60 years. Vaxigrip Tetra met EMA criteria in both age groups (as indicated by the dotted thick black line) [83].

This study also confirmed lot-to-lot equivalence (statistical equivalence of post-vaccination HI GMTs among the three QIV lots) and the non-inferiority of HI titers for the A strains and for the B strain when the comparator TIV was present; furthermore, the antibody response to the B strain lineage was even higher when the comparator TIV was absent (Figure 2). These results met EMA criteria for the licensing of influenza vaccines [67] and they were in line with those obtained from a previous study on younger adults immunized with the 2011–2012 formulation [85]. Investigating the patients 66–80 years, the analysis of HI antibody responses revealed that seroconversion rates and geometric mean increase met EMA criteria in adults aged 66–80 years for all the strains evaluated, although elevated seroprotection was observed on D0. These data strongly support the use of Vaxigrip Tetra in older adults (60–80 years), too, who constitute one of the highest-risk groups [93].

Good protection towards all four strains was also achieved in subjects presenting high-risk conditions; furthermore, seroprotection rates remained high one year after vaccination (≥98% in younger adults and ≥90% in older adults) [83].

In an investigation of SN responses conducted in a subset of vaccines according to EMA guidelines on influenza vaccines [94], a strong correlation was found between SN titers and protection against all four vaccine strains and between SN and HI antibody titers [83]. Concerning the safety profile, in agreement with previous studies [85,86,95], Vaxigrip Tetra proved to be well tolerated and displayed a similar safety profile to that of Vaxigrip [83]. In greater detail, solicited reactions, represented principally by injection-site pain, headache, malaise, and myalgia, were mainly grade 1 and resolved within 3 days. In both TIV and QIV vaccines, older adults showed lower solicited reaction rates than younger adults. Any vaccination-related SAE was notified [83].

#### 4.1.6. GQM07 Clinical Trial in Adults

An observer-blind, randomized, controlled, multicenter trial compared the immunogenicity and safety of the Northern Hemisphere 2015–2016 formulations of Vaxigrip Tetra with those of the licensed TIV in 300 Korean adults aged 18–60 years (GQM07; ClinicalTrials.gov No. NCT02550197) [84]. In that study, too, elevated pre-existing antibodies were observed in most participants, and increased by at least 4-fold upon QIV vaccination. The elevated immunogenicity measured by means of HI assay revealed that the ratio between the post-/pre-vaccination geometric mean of HI titers was ≥3.97, with a seroconversion rate of ≥40% for all strains with the exception A/H1N1 (39.7%). Equivalent results were obtained for the three strains contained in TIV. The immunogenicity of QIV towards the alternative B strain (Victoria) was superior to that of TIV. QIV also showed an acceptable safety profile, similar to that of TIV. More specifically, injection-site pain, myalgia, malaise, and headache represented the most common solicited reactions to QIV and TIV. Solicited reactions were mainly grade 1, and few participants developed grade 3 (severe) reactions (<2% per reaction type). All solicited reactions resolved within 5 days or less [84].

#### 4.1.7. GQM05 Clinical Trial in Children (6–35 Months)

Data regarding the clinical efficacy of Vaxigrip Tetra in unvaccinated children between 6 and 35 months of age were recently obtained from a large-scale, placebo-controlled phase III trial (GQM05, EudraCT: 2013-001231-51) performed in Europe, Asia, Latin America and South Africa, and presented at the Sixth ESWI Influenza Conference (2017) in Riga, Latvia [87]. The study was requested by EU health authorities as part of pediatric development. The efficacy trial in the youngest children aimed to reinforce the product profile and to demonstrate the efficacy of the product in a highly vulnerable population.

A total of 5805 children who had never been immunized were enrolled from March 2014 to December 2015. Children were stratified to include equal numbers of subjects immunized with 2 doses 0.5 mL of Vaxigrip Tetra (*n* = 2721) or placebo (*n* = 2715); in addition, 369 subjects received NH 2014–2015 TIV_YAM_ or TIV_VIC_. Influenza cases were confirmed through viral culture and reverse transcription polymerase chain reaction (RT-PCR). Severe laboratory-confirmed influenza illness was associated with at least one of the following symptoms: fever >39.5 °C in children aged <24 months or ≥39.0 °C in those aged ≥24 months, and/or at least one significant influenza like illness (ILI) symptom that prevented daily activity (cough, nasal congestion, rhinorrhea, pharyngitis, otitis, vomiting, diarrhea), and/or associated to one of the following events: acute otitis media (AOM), acute lower respiratory infection (ALRI). In this age-group, too, QIV promoted elevated immune responses towards the 4 strains included in the vaccine, thereby highlighting the broader protection against both B lineages offered by QIV in comparison with TIV.

Vaxigrip Tetra was protective towards laboratory-confirmed ILI caused not only by strains similar to the vaccine strains, but also by any circulating strains, supporting the conviction that the vaccine can also be used in this age-group.

As an additional medical benefit, QIV provided protection against severe influenza illness and reduced the risk of influenza-associated AOM and ALRI. QIV also reduced other relevant clinical outcomes in children, including medical examinations (2.3% in the QIV group vs. 5.6% in the placebo group) and antibiotic use (1.7% in the QIV group vs. 4.2% in the placebo group). QIV had a similar safety profile to those of TIV and placebo. No clinical concern was observed; QIV proved safe and well tolerated in unvaccinated children aged 6–35 months [87].

## 5. Conclusions

The current virological situation and the difficulty in predicting which influenza B virus lineage will be prevalent during the next influenza season have reduced immunological protection among vaccinated individuals. Thus, it is evident that new immunization strategies are required; replacing TIV with QIV could be an effective strategy, as suggested by the European Centre for Disease Prevention and Control (ECDC) [96]. In this regard, QIV licensure can meet this specific immunization need, and may be able to reduce influenza morbidity and influenza-associated mortality. The two groups that could benefit most from QIVs may be children and adolescents, who are significantly affected by influenza B viruses, and the elderly [32]. Chan’s group estimated that 60 out of every 1000 influenza cases could be prevented by using QIV instead of TIV [32]. An estimate of the public health and economic impact of QIV in comparison with TIV was performed in Europe by Uhart and colleagues [97]; this revealed that the use of QIV could considerably reduce both influenza cases and influenza-related costs (evaluated as €15 million and €77 million saved from general practitioners’ consultations and hospitalizations, respectively). Since 1968, the most widely-used influenza vaccine has been the corresponding TIV, over 1.8 billion doses of which have been distributed in more than 120 countries. This is the only influenza vaccine whose immunogenicity and clinical outcomes have been thoroughly evaluated, even in pregnant women at any stage of pregnancy and their infants. Regarding the use of Vaxigrip Tetra in pregnant women, a controlled randomized trial (GQM14; EudraCT: 2016-004763-40) is currently ongoing in Finland with the aim to evaluate its immunogenicity and safety (in terms of pregnancy and birth outcomes) in this high-risk group for influenza infection [98]. The quadrivalent formulation, Vaxigrip Tetra, is well tolerated and has not given rise to any safety concerns.

If one additional B virus strain were included, Vaxigrip Tetra (QIV) could provide significantly higher protection than the previously available TIV. Indeed, it eliminates the possibility of seasonal mismatched B strains, as observed during the last decade [33] with regard to previous influenza vaccines, without compromising protection towards influenza A. The immunogenicity and safety of Vaxigrip Tetra has been widely investigated in all age-groups (children, adults, and elderly) and has recently received EU authorization for its use in infants over 6 months of age. Vaxigrip Tetra not only displays an immunogenicity profile similar to that of the corresponding TIV; it has also proved able to induce a superior immune response towards the additional strain, without affecting immunogenicity for the other three strains. By contrast, TIV has been shown to evoke limited cross-reactivity against the missing B-lineage strain. In addition, the safety profile of Vaxigrip Tetra is equivalent to that of the corresponding licensed TIV, and no clinical concerns have been noted. Investigations conducted in children aged >6 months and in the elderly strongly support extending the use of Vaxigrip Tetra to these high-risk groups, thereby significantly increasing protection against influenza.

## Figures and Tables

**Table 1:** Criteria established by EMA for the licensing of influenza vaccines.

Ema Criteria for Influenza Vaccine Licensing	
Seroconv. Rate or Sig. Incr. %	>30.00 ELD–>40.00 ADU
Mean Gmt Increase	>2 ELD–>2.5 ADU
Seroprotection Rate %	>60.00 ELD–>70.00 ADU

Abbreviations: ADU: Adults; ELD: Elderly; EMA: European Medicine Agency; Seroconv. rate or sig. incr.: Seroconversion rate or significant increase; GMT: Geometric Mean Titer.

**Table 2:** Phase III clinical trials conducted on Vaxigrip Tetra.

Phase III Clinical Trials on Vaxigrip Tetra									
Clinical Trial Registry No.	Study Design	Type of Study	Objectives	Age	Participants	Countries	Influenza Season	Results	Ref.
**GQM02** EudraCT No. 2011-005374-33	Randomized, double-blind, active-controlled, multi-center study	Pivotal investigations testing the QIV licensed	**Primary Objective**: -Non-inferior immunogenicity of QIV to each TIV for the shared strains **Secondary Objective** -Humoral immune response induced by QIV -Superior immunogenicity of QIV to each TIV for the alternate B strain-Safety	3–8 y	1242 participants immunized with: -QIV (*n* = 887 *), -TIV-1 with B/Vic (*n* = 181 *), -an investigational TIV-2 with B/Yam (*n* = 174 *) * 65 subjects in the QIV group and 31 in the TIV groups were at-risk condition	Finland, Poland, Mexico and Taiwan	2013/2014 NH	Comparable immunogenicity of QIV vs. TIVs for the shared strains, and superiority for the additional B strain. Safety of QIV. Comparable immune responses induced by QIV in at-risk subjects with underlying comorbidity respect to the overall population after vaccination with QIV	[82]
**GQM09** WHO Universal Trial No. U1111-1127-7693	Open-label, uncontrolled	Pivotal investigations testing the QIV licensed	**Primary Objective**: -Immunogenicity -Safety	9–17 y	100 subjects immunized with: QIV	Taiwan	2013–2014 NH	Immunogenicity and safety demonstrated in children/adolescents	[81]
**GQM11** EudraCT No. 2014-000785-21	Randomized, multicenter, double-blinded for the QIV and TIV-2 groups, single-blinded for the TIV-1 group	Pivotal investigations testing the QIV licensed	**Primary Objective**: -Similar immunogenicity of 3 QIV lots -Non-inferiority of QIV respect to TIV for the shared strains **Secondary Objective** -Superior immunogenicity of QIV respect to TIV for the additional strain in each age group -Safety	≥18 y	2225 subjects (1114 adults (18–60 y) and 1111 subjects (>60 y) randomized 2:2:2:1:1 to receive: -a single dose of one of three lots of QIV, -the licensed TIV with B/Yam, -an investigational TIV with B/Vic	Belgium, France, Germany, Poland	2014–2015 NH	Lot-to-lot consistency demonstrated. Non-inferiority demonstrated between QIV and TIV. Higher immunogenicity of QIV against alternate B strains. Similar safety profile of QIV as the licensed TIV Comparable response in at-risk subjects	[83]
**GQM07** NCT02550197	Observer-blind, randomized, controlled, multicenter	Evaluation of the QIV licensed	**Primary Objective**: -Immunogenicity -Safety	18–60 y	300 subjects immunized with: -QIV (*n* = 200) -TIV (*n* = 100)	Republic of Korea	2015–2016 NH	Immunogenicity and safety demonstrated in adults	[84]
**GQM04** NCT01481454	Randomized, active-controlled, multicenter, double-blind for QIV lots, open-label for QIV vs. TIV	Supportive testing QIV batches produced with the TIV process	**Primary Objective**: -Safety of QIV and TIV **Secondary Objective** -Immunogenicity of QIV accordingly with EMA criteria -Immunogenic equivalence of three lots of QIV -Immunogenicity in children/adolescents	9–60 y	2090 subjects: -QIV (*n* = 1977) (1648 adults and 329 children/adolescents) -TIV (*n* = 111) (56 adults and 55 children/adolescents)	Australia, Philippines	2011/2012 NH and 2012 SH	Lot-to-lot consistency demonstrated. EMA criteria met in 9–60 years old. Comparable response and safety profiles of QIV to TIV	[86]
**GQM01** EudraCT: 2011-001976-21	Randomized, active-controlled, multicenter	Supportive testing QIV batches produced with the TIV process	**Primary Objective**: -Non-inferiority of QIV respect to a licensed TIV and an investigational TIV **Secondary Objective** -Safety -Superior ab response to each B strain in the QIV group respect to the corresponding B strain in TIV group lacking this strain	≥18 y	1565 participants: -QIV (*n* = 1116 subjects), -licensed TIV with B/Vic (*n* = 226), -the investigational TIV with B/Yam (*n* = 223)	France, Germany	2011–2012 NH	Safety of QIV and superior immunogenicity of QIV respect TIV for the unmatched strains and non-inferiority for the matched strains	[85]
**GQM05** EudraCT: 2013-001231-51	Randomized, double-blind (except TIV groups open-label), placebo-controlled, multicenter	Pediatric investigation	**Primary Objective**: -Efficacy to prevent at least one of the following endpoints: • laboratory-confirmed influenza illness due to any influenza circulating strain (A or B types) • laboratory-confirmed influenza illness caused by influenza strains similar to those contained in the vaccine **Secondary Objective** -Non-inferiority of QIV vs. TIV as well as superior immunogenicity for B strains -Safety	6–35 m	5805 participants: -QIV (*n* = 2721 subjects), -placebo (*n* = 2715), -licensed TIV with B/Vic or B/Yam (*n* = 369)	Europe, Asia, Latin America, South Africa	2014–2015 NH TIV_YAM_ or TIV_VIC_	Preliminary results: Immunogenicity of QIV Prevention of influenza caused by strains similar to the vaccine strains and any circulating strains Protection against severe influenza illness and reduced the risk for AOM and ALRI associated with influenza Reduction of other relevant clinical outcomes for children, including medical visits and antibiotic use Safety	[87]

Abbreviations: Ab: antibody; EMA: European Medicine Agency; m: months; NH: Northern hemisphere; SH: Southern hemisphere; Vic: Victoria; y: years; Yam: Yamagata; *: subjects at-risk condition.

## References

[1] Weekly Epidemiological Record. http://www.who.int/wer/2012/wer8747.pdf?ua=1&ua=1.

[2] Kocik J., Kołodziej M., Joniec J., Kwiatek M., Bartoszcze M. (2014). Antiviral activity of novel oseltamivir derivatives against some influenza virus strains. Acta Biochim. Pol..

[3] Coughlan L., Lambe T. (2015). Measuring Cellular Immunity to Influenza: Methods of Detection, Applications and Challenges. Vaccines.

[4] Esposito S., Molteni C.G., Daleno C., Valzano A., Fossali E., Da Dalt L., Cecinati V., Bruzzese E., Giacchino R., Giaquinto C. (2011). Clinical and socioeconomic impact of different types and subtypes of seasonal influenza viruses in children during influenza seasons 2007/2008 and 2008/2009. BMC Infect. Dis..

[5] Centers for Disease Control and Prevention (2010). Estimates of deaths associated with Seasonal Influenza—United States, 1976–2007. Morb. Mortal. Wkly. Rep..

[6] Gianchecchi E., Trombetta C., Piccirella S., Montomoli E. (2016). Evaluating influenza vaccines: Progress and perspectives. Future Virol..

[7] Heo J.Y., Song J.Y., Noh J.Y., Choi M.J., Yoon J.G., Lee S.N., Cheong H.J., Kim W.J. (2017). Effects of influenza immunization on pneumonia in the elderly. Hum. Vaccines Immunother..

[8] Siriwardena A.N. (2012). Increasing evidence that influenza is a trigger for cardiovascular disease. J. Infect. Dis..

[9] Influenza ACIP Vaccine Recommendations. www.cdc.gov/vaccines/hcp/acip-recs/vacc-specific/flu.html.

[10] Background Paper on Influenza Vaccines and Immunization SAGE Working Group. http://www.who.int/immunization/sage/meetings/2012/april/1_Background_Paper_Mar26_v13_cleaned.pdf.

[11] Fiore A.E., Uyeki T.M., Broder K., Finelli L., Euler G.L., Singleton J.A., Iskander J.K., Wortley P.M., Shay D.K., Bresee J.S. (2010). Prevention and control of influenza with vaccines: Recommendations of the Advisory Committee on Immunization Practices (ACIP), 2010. MMWR Recomm. Rep..

[12] Reperant L.A., Grenfell B.T., Osterhaus A.D. (2015). Quantifying the risk of pandemic influenza virus evolution by mutation and re-assortment. Vaccine.

[13] Nobusawa E., Sato K. (2006). Comparison of the mutation rates of human influenza A and B viruses. J. Virol..

[14] Irving S.A., Patel D.C., Kieke B.A., Donahue J.G., Vandermause M.F., Shay D.K., Belongia E.A. (2012). Comparison of clinical features and outcomes of medically attended influenza A and influenza B in a defined population over four seasons: 2004–2005 through 2007–2008. Influenza Other Respir. Viruses.

[15] Tafalla M., Buijssen M., Geets R., Vonk Noordegraaf-Schouten M. (2016). A comprehensive review of the epidemiology and disease burden of Influenza B in 9 European countries. Hum. Vaccines Immunother..

[16] Influenza (Seasonal). http://www.who.int/mediacentre/factsheets/fs211/en/.

[17] Petrova V.N., Russell C.A. (2018). The evolution of seasonal influenza viruses. Nat. Rev. Microbiol..

[18] Barr I.G., Russell C., Besselaar T.G., Cox N.J., Daniels R.S., Donis R., Engelhardt O.G., Grohmann G., Itamura S., Kelso A. (2014). WHO recommendations for the viruses used in the 2013–2014 Northern Hemisphere influenza vaccine: Epidemiology, antigenic and genetic characteristics of influenza A(H1N1) pdm09, A(H3N2) and B influenza viruses collected from October 2012 to January 2013. Vaccine.

[19] Klimov A.I., Garten R., Russell C., Barr I.G., Besselaar T.G., Daniels R., Engelhardt O.G., Grohmann G., Itamura S., Kelso A. (2012). WHO recommendations for the viruses to be used in the 2012 Southern Hemisphere influenza vaccine: Epidemiology, antigenic and genetic characteristics of influenza A(H1N1)pdm09, A(H3N2) and B influenza viruses collected from February to September 2011. Vaccine.

[20] Ampofo W.K., Baylor N., Cobey S., Cox N.J., Daves S., Edwards S., Ferguson N., Grohmann G., Hay A., WHO Writing Group (2012). Improving influenza vaccine virus selection: Report of a WHO informal consultation held at WHO headquarters, Geneva, Switzerland, 14–16 June 2010. Influenza Other Respir. Viruses.

[21] Peltola V., Ziegler T., Ruuskanen O. (2003). Influenza A and B virus infections in children. Clin. Infect. Dis..

[22] Rota P.A., Wallis T.R., Harmon M.W., Rota J.S., Kendal A.P., Nerome K. (1990). Cocirculation of two distinct evolutionary lineages of influenza type B virus since 1983. Virology.

[23] Chen J.M., Guo Y.J., Wu K.Y., Guo J.F., Wang M., Dong J., Zhang Y., Li Z., Shu Y.L. (2007). Exploration of the emergence of the Victoria lineage of influenza B virus. Arch. Virol..

[24] Shaw M.W., Xu X., Li Y., Normand S., Ueki R.T., Kunimoto G.Y., Hall H., Klimov A., Cox N.J., Subbarao K. (2002). Reappearance and global spread of variants of influenza B/Victoria/2/87 lineage viruses in the 2000–2001 and 2001–2002 seasons. Virology.

[25] Hannoun C. (2013). The evolving history of influenza viruses and influenza vaccines. Expert Rev. Vaccines.

[26] Centers for Disease Control and Prevention (CDC) (2008). Influenza activity—United States and worldwide, 2007–08 season. Morb. Mortal. Wkly. Rep..

[27] Caini S., Huang Q.S., Ciblak M.A., Kusznierz G., Owen R., Wangchuk S., Henriques C.M., Njouom R., Fasce R.A., Yu H. (2015). Epidemiological and virological characteristics of influenza B: Results of the Global Influenza B Study. Influenza Other Respir Viruses.

[28] Ambrose C.S., Levin M.J. (2012). The rationale for quadrivalent influenza vaccines. Hum. Vaccines. Immunother..

[29] Belshe R.B. (2010). The need for quadrivalent vaccine against seasonal influenza. Vaccine.

[30] Influenza Virus Characterisation. https://ecdc.europa.eu/sites/portal/files/media/en/publications/Publications/influenza-virus-characterisation-may-2016.pdf.

[31] Heikkinen T., Ikonen N., Ziegler T. (2014). Impact of influenza B lineagelevel mismatch between trivalent seasonal influenza vaccines and circulating viruses, 1999–2012. Clin. Infect. Dis..

[32] Chan P.K., Chan M.C., Cheung J.L., Lee N., Leung T.F., Yeung AC., Wong M.C., Ngai K.L., Nelson E.A., Hui D.S. (2013). Influenza B lineage circulation and hospitalization rates in a subtropical city, Hong Kong, 2000–2010. Clin. Infect. Dis..

[33] Belshe R.B., Coelingh K., Ambrose C.S., Woo J.C., Wu X. (2010). Efficacy of live attenuated influenza vaccine in children against influenza B viruses by lineage and antigenic similarity. Vaccine.

[34] Tricco A.C., Chit A., Soobiah C., Hallett D., Meier G., Chen M.H., Tashkandi M., Bauch C.T., Loeb M. (2013). Comparing influenza vaccine efficacy against mismatched and matched strains: A systematic review and meta-analysis. BMC Med..

[35] De Jong J.C., Beyer W.E., Palache A.M., Rimmelzwaan G.F., Osterhaus A.D. (2000). Mismatch between the 1997/1998 influenza vaccine and the major epidemic A(H3N2) virus strain as the cause of an inadequate vaccine-induced antibody response to this strain in the elderly. J. Med. Virol..

[36] Vaccines and Related Biological Products Advisory Committee. https://www.fda.gov/ohrms/dockets/ac/07/transcripts/2007-4282t2.htm.

[37] Lee B.Y., Bartsch S.M., Willig A.M. (2012). The economic value of a quadrivalent versus trivalent influenza vaccine. Vaccine.

[38] De Boer P.T., Crépey P., Pitman R.J., Macabeo B., Chit A., Postma M.J. (2016). Cost-Effectiveness of Quadrivalent versus Trivalent Influenza Vaccine in the United States. Value Health.

[39] Flannery B., Clippard J., Zimmerman R.K., Nowalk M.P., Jackson M.L., Jackson L.A., Monto A.S., Petrie J.G., McLean H.Q., Belongia E.A. (2015). Early estimates of seasonal influenza vaccine effectiveness- United States, January 2015. Morb. Mortal. Wkly. Rep..

[40] People at High Risk of Developing Flu–Related Complications. https://www.cdc.gov/flu/about/disease/high_risk.htm>.

[41] Wang B., Russell M.L., Brewer A., Newton J., Singh P., Ward B.J., Loeb M. (2017). Single radial haemolysis compared to haemagglutinin inhibition and microneutralization as a correlate of protection against influenza A H3N2 in children and adolescents. Influenza Other Respir. Viruses.

[42] Russell S., McCahon D., Beare A. (1975). A single radial haemolysis technique for the measurement of influenza antibody. J. Gen. Virol..

[43] Hirst G.K. (1942). Adsorption of influenza hemagglutinins and virus by red blood cells. J. Exp. Med..

[44] Salk J.E. (1944). A simplified procedure for titrating hemagglutinating capacity of influenza virus and the corresponding antibody. J. Immunol..

[45] Schild G., Pereira M., Chakraverty P. (1975). Single-radial-haemolysis: A new method for the assay of antibody to influenza haemagglutinin: Applications for diagnosis and seroepidemiologic surveillance of influenza. Bull. World Health Organ..

[46] Trombetta C.M., Montomoli E. (2016). Influenza immunology evaluation and correlates of protection: A focus on vaccines. Expert Rev. Vaccines.

[47] Trombetta C.M., Perini D., Vitale L., Cox R.J., Stanzani V., Piccirella S., Montomoli E. (2015). Validation of Single Radial Haemolysis assay: A reliable method to measure antibodies against influenza viruses. J. Immunol. Methods.

[48] Wilson G., Ye Z., Xie H., Vahl S., Dawson E., Rowlen K. (2017). Automated interpretation of influenza hemagglutination inhibition (HI) assays: Is plate tilting necessary?. PLoS ONE.

[49] Wood J.M., Gaines-Das R.E., Taylor J., Chakraverty P. (1994). Comparison of influenza serological techniques by international collaborative study. Vaccine.

[50] World Health Organization (2011). Manual for the Laboratory Diagnosis and Virological Surveillance of Influenza.

[51] Products CFPM (1997). Note for Guidance on Harmonisation of Requirements for Influenza Vaccines.

[52] Cox R.J. (2013). Correlates of protection to influenza virus, where do we go from here?. Hum. Vaccines Immunother..

[53] Levy O. (2007). Innate immunity of the newborn: Basic mechanisms and clinical correlates. Nat. Rev. Immunol..

[54] Saso A., Kampmann B. (2017). Vaccine responses in newborns. Semin. Immunopathol..

[55] Walter E.B., Rajagopal S., Zhu Y., Neuzil K.M., Fairchok M.P., Englund J.A. (2010). Trivalent inactivated influenza vaccine (TIV) immunogenicity in children 6 through 23 months of age: Do children of all ages respond equally?. Vaccine.

[56] Levandowski R.A., Regnery H.L., Staton E., Burgess B.G., Williams M.S., Groothuis J.R. (1991). Antibody responses to influenza B viruses in immunologically unprimed children. Pediatrics.

[57] Siegrist C.A., Aspinall R. (2009). B-cell responses to vaccination at the extremes of age. Nat. Rev. Immunol..

[58] Del Giudice G., Goronzy J.J., Grubeck-Loebenstein B., Lambert P.H., Mrkvan T., Stoddard J.J., Doherty T.M. (2017). Fighting against a protean enemy: Immunosenescence, vaccines, and healthy aging. NPJ Aging Mech. Dis..

[59] Sasaki S., Sullivan M., Narvaez C.F., Holmes T.H., Furman D., Zheng N.Y., Nishtala M., Wrammert J., Smith K., James J.A. (2011). Limited efficacy of inactivated influenza vaccine in elderly individuals is associated with decreased production of vaccine-specific antibodies. J. Clin. Investig..

[60] Jefferson T., Rivetti D., Rivetti A., Rudin M., Di Pietrantonj C., Demicheli V. (2005). Efficacy and effectiveness of influenza vaccines in elderly people: A systematic review. Lancet.

[61] Beyer W.E., McElhaney J., Smith D.J., Monto A.S., Nguyen-Van-Tam J.S., Osterhaus A.D. (2013). Cochrane rearranged: Support for policies to vaccinate elderly people against influenza. Vaccine.

[62] Mysliwska J., Trzonkowski P., Szmit E., Brydak L.B., Machala M., Mysliwski A. (2004). Immunomodulating effect of influenza vaccination in the elderly differing in health status. Exp. Gerontol..

[63] Zhang Y., Wallace D.L., de Lara C.M., Ghattas H., Asquith B., Worth A., Griffin G.E., Taylor G.P., Tough D.F., Beverley P.C. (2007). In vivo kinetics of human natural killer cells: The effects of ageing and acute and chronic viral infection. Immunology.

[64] Goronzy J.J., Fulbright J.W., Crowson C.S., Poland G.A., O’Fallon W.M., Weyand C.M. (2001). Value of immunological markers in predicting responsiveness to influenza vaccination in elderly individuals. J. Virol..

[65] Saurwein-Teissl M., Lung T.L., Marx F., Gschösser C., Asch E., Blasko I., Parson W., Böck G., Schönitzer D., Trannoy E. (2002). Lack of antibody production following immunization in old age: Association with CD8(+)CD28(-) T cell clonal expansions and an imbalance in the production of Th1 and Th2 cytokines. J. Immunol..

[66] Allman D., Miller J.P. (2005). B cell development and receptor diversity during aging. Curr. Opin. Immunol..

[67] Haynes L., Eaton S.M. (2005). The effect of age on the cognate function of CD4+ T cells. The frequencies of CD8+ CD28− T cells can be used to predict influenza vaccine response in elderly people. Immunol. Rev..

[68] Frasca D., Riley R.L., Blomberg B.B. (2005). Humoral immune response and B-cell functions including immunoglobulin class switch are downregulated in aged mice and humans. Semin. Immunol..

[69] Wijnans L., Voordouw B. (2016). A review of the changes to the licensing of influenza vaccines in Europe. Influenza Other Respir. Viruses.

[70] McElhaney J.E., Xie D., Hager W.D., Barry M.B., Wang Y., Kleppinger A., Ewen C., Kane K.P., Bleackley R.C. (2006). T cell responses are better correlates of vaccine protection in the elderly. J. Immunol..

[71] Furman D., Davis M.M. (2015). New approaches to understanding the immune response to vaccination and infection. Vaccine.

[72] Leng S.X., McElhaney J.E., Walston J.D., Xie D., Fedarko N.S., Kuchel G.A. (2008). ELISA and multiplex technologies for cytokine measurement in inflammation and aging research. J. Gerontol. A Biol. Sci. Med. Sci..

[73] Czerkinsky C.C., Nilsson L.A., Nygren H., Ouchterlony O., Tarkowski A. (1983). A solid-phase enzyme-linked immunospot (ELISPOT) assay for enumeration of specific antibody-secreting cells. J. Immunol. Methods.

[74] Domachowske J.B., Pankow-Culot H., Bautista M., Feng Y., Claeys C., Peeters M., Innis B.L., Jain V. (2013). A randomized trial of candidate inactivated quadrivalent influenza vaccine versus trivalent influenza vaccines in children aged 3–17 years. J. Infect. Dis..

[75] Pyhala R., Kleemola M., Kumpulainen V., Vartiainen E., Lappi S., Ponka A., Cantell K. (1992). Immune response to inactivated influenza virus vaccine: Antibody reactivity with epidemic influenza B viruses of two highly distinct evolutionary lineages. Vaccine.

[76] Baldo V., Baldovin T., Floreani A., Fragapane E., Trivello R., Family Medicine G. (2007). Response of influenza vaccines against heterovariant influenza virus strains in adults with chronic diseases. J. Clin. Immunol..

[77] Camilloni B., Neri M., Lepri E., Iorio A.M. (2009). Cross-reactive antibodies in middle-aged and elderly volunteers after MF59-adjuvanted subunit trivalent influenza vaccine against B viruses of the B/Victoria or B/Yamagata lineages. Vaccine.

[78] Gresset-Bourgeois V., Leventhal P.S., Pepin S., Hollingsworth R., Kazek-Duret M.P., De Bruijn I., Samson S.I. (2017). Quadrivalent inactivated influenza vaccine (VaxigripTetra™). Expert Rev. Vaccines.

[79] Vaxigrip Tetra. http://mri.cts-mrp.eu/Human/Product/Details/47992.

[80] Vaxigrip Tetra Quadrivalent Influenza Vaccine (Split Virion, Inactivated). https://mri.ctsmrp.eu/Human/Downloads/DE_H_1949_001_PAR.pdf.

[81] Lu C.Y., Ferracin C., Chiu C.H., Lavis N., Huang C.H., Huang L.M. (2016). Immunogenicity and safety of a quadrivalent influenza vaccine in children and adolescents in Taiwan: A phase III open-label trial. Trials Vaccinol..

[82] Pepin S., Szymanski H., Rochín Kobashi I.A., Villagomez Martinez S., González Zamora J.F., Brzostek J., Huang L.M., Chiu C.H., Chen P.Y., Ahonen A. (2016). Safety and immunogenicity of an intramuscular quadrivalent influenza vaccine in children 3 to 8 y of age: A phase III randomized controlled study. Hum. Vaccines Immunother..

[83] Sesay S., Brzostek J., Meyer I., Donazzolo Y., Leroux-Roels G., Rouzier R., Astruc B., Szymanski H., Toursarkissian N., Vandermeulen C. (2017). Safety, immunogenicity, and lot-to-lot consistency of a split-virion quadrivalent influenza vaccine in younger and older adults: A phase III randomized, double-blind clinical trial. Hum. Vaccines Immunother..

[84] Choi W.S., Noh J.Y., Lee J., Choi J.Y., Lee J.S., Kim M.S., Kim H.S., Bang J., Lavis N., Kim W.J. (2017). Immunogenicity and safety of a split-virion quadrivalent influenza vaccine in adults 18–60 years of age in the Republic of Korea. Hum. Vaccines Immunother..

[85] Pépin S., Donazzolo Y., Jambrecina A., Salamand C., Saville M. (2013). Safety and immunogenicity of a quadrivalent inactivated influenza vaccine in adults. Vaccine.

[86] Cadorna-Carlos J.B., Nolan T., Borja-Tabora C.F., Santos J., Montalban M.C., de Looze F.J., Eizenberg P., Hall S., Dupuy M., Hutagalung Y. (2015). Safety, immunogenicity, and lot-to-lot consistency of a quadrivalent inactivated influenza vaccine in children, adolescents, and adults: A randomized, controlled, phase III trial. Vaccine.

[87] Pepin S., Dupuy M., Borja-Tabora C., Montellano M., Bravo L., Cadorna-Carlos J., Santos J., De Castro J.-A., Rivera-Medina D.M., Cutland C. Efficacy, immunogenicity and safety of a quadrivalent inactivated influenza vaccine in children from 6 to 35 months. Proceedings of the Sixth ESWI Influenza Conference.

[88] Clinical Safety Data Management: Definitions and Standards for Expedited Reporting E2A. https://www.ich.org/fileadmin/Public_Web_Site/ICH_Products/Guidelines/Efficacy/E2A/Step4/E2A_Guideline.pdf.

[89] Trombetta C.M., Gianchecchi E., Montomoli E. (2018). Influenza vaccines: Evaluation of the safety profile. Hum. Vaccines Immunother..

[90] Goodwin K., Viboud C., Simonsen L. (2006). Antibody response to influenza vaccination in the elderly: A quantitative review. Vaccine.

[91] McElhaney J.E., Zhou X., Talbot H.K., Soethout E., Bleackley R.C., Granville D.J., Pawelec G. (2012). The unmet need in the elderly: How immunosenescence, CMV infection, co-morbidities and frailty are a challenge for the development of more effective influenza vaccines. Vaccine.

[92] Clinical Trial Results: Immunogenicity and Lot-to-Lot Consistency Study of a Quadrivalent Influenza Vaccine in Adult and Elderly Subjects. https://www.clinicaltrialsregister.eu/ctr-search/trial/2014-000785-21/results.

[93] Pasteur S. (2018). Personal communication.

[94] Guideline on Influenza Vaccines. http://www.ema.europa.eu/docs/en_GB/document_library/Scientific_guideline/2016/07/WC500211324.pdf.

[95] Moa A.M., Chughtai A.A., Muscatello D.J., Turner R.M., MacIntyre C.R. (2016). Immunogenicity and safety of inactivated quadrivalent influenza vaccine in adults: A systematic review and meta analysis of randomised controlled trials. Vaccine.

[96] Risk Assessment for Seasonal Influenza, EU/EEA, 2017–2018. https://ecdc.europa.eu/sites/portal/files/documents/RRA%20seasonal%20influenza%20EU%20EEA%202017-2018.pdf.

[97] Uhart M., Bricout H., Clay E., Largeron N. (2016). Public health and economic impact of seasonal influenza vaccination with quadrivalent influenza vaccines compared to trivalent influenza vaccines in Europe. Hum. Vaccines Immunother..

[98] Clinical Trials for Quadrivalent Vaccine Pregnant Women. https://www.clinicaltrialsregister.eu/ctr-search/search?query=quadrivalent+vaccine+pregnant+women.

